# Management of Pediatric Superficial Partial-Thickness Burns with Polyhexamethylene Biguanide: Outcomes and Influencing Factors

**DOI:** 10.3390/jcm13113074

**Published:** 2024-05-24

**Authors:** Aba Lőrincz, Hermann Nudelman, Anna Gabriella Lamberti, András Garami, Krisztina Adrienne Tiborcz, Tamás Zoltán Kovács, Gergő Józsa

**Affiliations:** 1 Department of Thermophysiology, Institute for Translational Medicine, Medical School, University of Pécs, 12 Szigeti Street, H7624 Pécs, Hungary; aba.lorincz@gmail.com (A.L.); nuhwaao.pte@tr.pte.hu (H.N.); lamberti.anna@pte.hu (A.G.L.); andras.garami@aok.pte.hu (A.G.); 2 Division of Pediatric Surgery, Traumatology, Urology and Pediatric Otolaryngology, Department of Pediatrics, Medical School, University of Pécs, 7 József Attila Street, H7623 Pécs, Hungary; 3 Pediatric Surgery Unit, Szent-Györgyi Albert Pediatric Clinic and Children’s Health Center, University of Szeged, 14–15 Korányi Avenue, H6721 Szeged, Hungary; krisztinatiborcz@gmail.com (K.A.T.); kovacs.tamas@med.u-szeged.hu (T.Z.K.)

**Keywords:** pediatric, child, partial-thickness, second-degree, burn, PHMB, Polyhexanide, Polyhexamethylen Biguanide

## Abstract

**Background:** In burn care, achieving swift healing with minimal complications remains paramount. This investigation assesses the role of polyhexamethylene biguanide (PHMB) in managing pediatric superficial partial-thickness burns, focusing on the effects of various patient-specific factors on recovery. **Methods:** Through a retrospective analysis of 27 pediatric cases treated with PHMB, we evaluated the impact of age, burn size, dressing frequency, treatment delay, cold therapy application, and analgesic usage on the time until reepithelialization (TTRE). **Results:** The majority of patients benefited from early cold therapy, yet only 1 in 3 patients received analgesics. A mean healing time of 8.78 (SD: 2.64) days was observed, with the extent of the burn showing a strong correlation (r: 0.63) to TTRE. Most treatments were managed outpatient, evidenced by a negligible average hospital stay (0.96 days), with recorded no complications. **Conclusions:** Our findings endorse PHMB as a promising treatment for superficial second-degree burns in young patients, due to the observed stable and rapid wound closure without the association of increased risks. Continued exploration into the optimal application of prehospital interventions and the comprehensive benefits of PHMB in pediatric burn management is necessary. Future research should assess long-term outcomes, including functionality, scar quality, and patient satisfaction.

## 1. Introduction

Burn injuries represent a significant global health issue, disproportionately affecting pediatric populations in the African region. Worldwide and in the Pan-American area, they rank as the third leading cause of accidental injury and death in children aged 1 to 4 years, and the second in Europe, highlighting the critical need for effective burn care strategies [1]. Unique physiological attributes of children, including their thinner skin and the rapid growth phase, exacerbate the risk of deep tissue damage, protracted healing, and significant scarring from burn injuries [2]. Among these, superficial partial-thickness (i.e., superficial second-degree or II/1) burns are characterized by damage to both the epidermis and the papillary layer of the dermis, necessitating a balanced approach to care [3,4]. Mixed-depth injuries contain islands of II/1 and deep partial-thickness (II/2) burns in a map-like pattern, penetrating the reticular dermis as well. These wounds not only demand acute medical intervention to facilitate their closure but also a holistic strategy to address potential functional, esthetic, psychological, and social impacts, underscoring the complexity of pediatric burn management [5].

Historically, the treatment of burns, particularly in children, has focused on preventing infection and promoting healing, due to their vulnerability to pathogens and the catastrophic consequences of microbial colonization in burn wounds. Traditional agents include silver sulfadiazine (SSD) and iodine-based products, which have been staples in burn care [6,7]. However, concerns over toxicity, resistance, and healing impairment have driven the search for alternative remedies.

Polyhexamethylene Biguanide (Polyhexanide or PHMB in short) is a polymer-based, broad-spectrum antimicrobial agent. It consists of repeating basic biguanide units linked by hexamethylene hydrocarbon chains, resulting in a cationic and amphipathic structure. PHMB exhibits potent activity against a plethora of pathogens including methicillin-resistant Staphylococcus aureus (MRSA), Pseudomonas aeruginosa, and Candida albicans—prevalent burn infections that can significantly delay healing processes [8]. There is minimal evidence of resistance development against PHMB, making it a sustainable option for long-term use in healthcare settings [9]. Beyond its germicide properties, it has been observed to support wound healing, possibly through establishing moist microenvironments and minimizing inflammation [10]. PHMB can be incorporated into various forms, such as gels, solutions, creams, and impregnated dressings, allowing for flexible application based on the needs of the wound [11].

In exploring strategies to address infection control and mitigate antimicrobial resistance, it is important to consider the mechanisms through which drugs exert their effects. Previous research has focused on PHMB, investigating its ability to preferentially target bacteria over host cells. Contrary to the conventional understanding that PHMB disrupts microbial membranes [12], a study has revealed its capability to penetrate bacterial cells, leading to inhibited cell division and DNA condensation [13]. It proposes an alternative mechanism of antimicrobial action, potentially involving DNA interaction. Further analysis demonstrated that PHMB forms nanoparticles upon interaction with bacterial chromosomal DNA, and its bacterial growth inhibitory effects can be attenuated by co-application with the DNA-binding agent Hoechst 33258, supporting the notion of a DNA-targeted antimicrobial mechanism. Interestingly, while PHMB is capable of entering mammalian cells, it appears to be sequestered within endosomes and prevented from accessing the nucleus, highlighting a differential impact on bacterial versus mammalian DNA [11]. These previous findings draw attention to a distinct antimicrobial mechanism by which PHMB selectively interacts with and condenses bacterial chromosomes. Particularly, the absence of reported resistance to PHMB suggests this mechanism could represent a promising avenue for antimicrobial development, circumventing common pathways of resistance [8,13]. 

While PHMB is encouraging, studies specifically targeting pediatric burn patients are limited, necessitating cautious extrapolation of data from adult and in vitro studies [14,15]. Although generally well-tolerated, PHMB can cause allergic contact dermatitis in some individuals, therefore, monitoring and patient-specific considerations are crucial [16]. PHMB usage in certain jurisdictions may face regulatory limitations or require specific approvals, impacting its availability and usage in pediatric burn care. Depending on the healthcare setting, the cost of PHMB formulations compared to traditional interventions may ultimately affect its adoption and widespread use. 

Scientific literature, while affirming the antimicrobial prowess of PHMB, seldom ventures into its direct implications on healing timelines or how it influences critical outcomes such as scar formation, functional impairment, or pain management. Moreover, the interaction of PHMB with patient-specific factors—age, Total Body Surface Area (TBSA) affected, frequency of dressing changes, and the interval between injury occurrence and treatment commencement—remains underexplored. These features are pivotal in the context of pediatric burns, where they can significantly dictate the trajectory of healing and recovery. For instance, the TBSA affected by burns, a critical measure of burn severity, has been directly linked to the risk of infection and length of hospital stay, thereby affecting healing outcomes [17].

Addressing this knowledge gap, our study thoroughly evaluates PHMB in pediatric II/1 burn management. We aim to investigate its influence on the time to reepithelialization (TTRE) while dissecting how this impact is modulated by intrinsic patient metrics and wound characteristics. Through this inquiry, we examine the multifaceted role of PHMB in pediatric burn care to encompass its overall healing capabilities, safety profile, and compatibility with broader treatment strategies.

## 2. Materials and Methods

### 2.1. Study Design and Setting

This retrospective, single-arm, multi-center cohort study was conducted in accordance with the Strengthening the Reporting of Observational Studies in Epidemiology (STROBE) guidelines [18]. The research setting was two Hungarian tertiary pediatric burn units (at the Universities of Pécs and Szeged. The study period spanned from 17 October 2023 to 15 February 2024, allowing for the evaluation of short-term and intermediate healing outcomes.

### 2.2. Participants

The cohort comprised 27 pediatric patients, aged 1 to 13 years, diagnosed with II/1 partial-thickness burns covering between 1 and 10% TBSA. Half of the patients were admitted in Pécs and the other half in Szeged. Patient inclusion criteria were: (1) age below 18 years, (2) confirmed diagnosis of II/1 burns, (3) treatment initiation within 24 h of injury, and (4) consent for PHMB application. Exclusion criteria included (1) known hypersensitivity to PHMB, (2) presence of full-thickness burns, (3) receiving skin grafts, (4) missing photo-documentation, (5) absence on follow-up examinations before complete healing, (6) and concurrent injuries or diseases requiring systemic interventions.

### 2.3. Intervention

All participants received sterile, preserved 0.04% PHMB gel (LAVANID^®^—Wound Gel, SERAG-WIESSNER, Naila, Germany) topical application as part of their wound care regimen. The treatment protocol entailed cleaning the wound with sterile saline, and bullectomy, followed by PHMB application and dressing with a versatile wound contact layer. We utilized a woven cotton paraffine-impregnated net (Grassolind^®^, HARTMANN GROUP, Heidenheim, Germany), or a similarly paraffine-impregnated, fine-meshed gauze, containing 0.5% chlorhexidine as well (Bactigras^®^, Smith + Nephew, Watford, UK). They are capable of covering large body surfaces without causing hypersensitive reactions. Adjunct medications, including analgesia with a synthetic opioid; 0.1–0.2 mg/kg nalbuphine injection (Nubain, ALTAMEDICS GmbH, Cologne, Germany), and sedation with midazolam (Dormicum, Egis Gyógyszergyár Zrt, Budapest, Hungary) were administered based on clinical indications and parental consent. Hospitalization is required in the cases of pediatric burns covering more than 10% TBSA—or ≥5% in very young children (<5 years old) because they have lower physiological reserves, and—may necessitate admission for proper pain management, hydration, and monitoring potential complications [19]. Burns on critical areas like the face, hands, genital region, or over major joints are more prone to entail hospital admission. Deeper than II/1 injuries usually require skin grafting after debridement, while chemical, electric, and irradiation burns, smoke inhalation, or injury of critical areas necessitate consultations with experts (such as orthopedic or hand surgeon, cardiologist, or ophthalmologist) and referral to specialized burn units due to the complexity of care needed [20].

### 2.4. Outcome Measures

TTRE was the primary endpoint, defined as the number of days from injury until wound closure. Complete wound closure (i.e., healing) was described as a new, continuous, shiny, layer of epithelium developing on the entire surface of the injury. Secondary outcomes included the incidence of wound infections, the number of dressing changes (thus, to get the total amount of dressings applied, the initial dressing has to be added to the number of reapplications), length of hospital stay (LOS), and any reported complications. Patient and wound metrics—age, sex, time since injury, prehospital treatments, burn depth, -area, and -location —, were recorded during the first meetings and summarized later in Microsoft Excel 2021 (Microsoft Corporation, Redmond, WA, USA). Injuries and their healing progression were photo-documented on the initial and each follow-up examination. No data point was missing for any of the included patients.

### 2.5. Statistical Evaluation

Descriptive statistics were calculated for all endpoints using counts (*n*), means, standard deviations (SD), medians, interquartile ranges (IQR), 25th percentiles (IQR25), 75th percentiles (IQR75), and ranges. Correlations of continuous variables were calculated via Pearson’s coefficient while continuous against discrete outcomes were computed with point-biserial correlation coefficients (*r*). Statistical comparison was conducted with Chi^2^- and Fischer’s Exact tests (in cases of categorical variables) and Mann-Whitney U test (when analyzing continuous outcomes) using Python 3.9.16. (Python Software Foundation, Wilmington, DE, USA) with numpy, pandas, and scikit-learn libraries. Differences were deemed significant when *p* ≤ 0.05. For the burn localization and etiology analyses, Kruskal-Wallis tests were used, due to non-normal distribution and small sample sizes. Linear regression, random forest, and deep learning models were trained to predict TTRE, utilizing Pytorch 2.0 (Linux Foundation, San Francisco, CA, USA). Their value was validated by mean squared errors (MSE) which indicates the average squared difference between the estimated values and the actual value of TTRE, with a lower MSE representing a better fit. Additionally, the R^2^ score, which can range from -∞ to 1, provides a measure of how well the observed outcomes are replicated by the model, based on the proportion of total variation of variables.

## 3. Results

**Figure 1:**
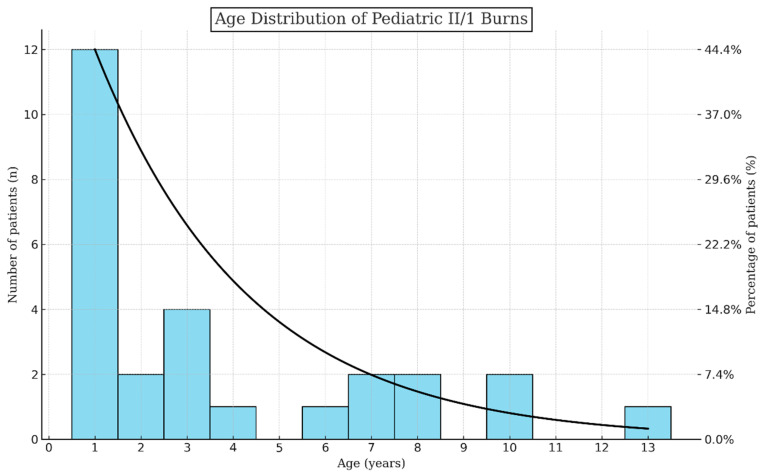
Age distribution of pediatric II/1 burns. A trendline shows the predicted age allotment over the measured ones visualized by the histogram.

Our study evaluated 27 pediatric patients—14 managed at Pécs and 13 at Szeged, Hungary—treated with PHMB gel for II/1 burns. This cohort was characterized by a male-predominant gender distribution (66% male, 33% female) and an age range from 1 to 13 years (mean: 3.74, SD: 4.73), similar to international reports [1,21]. An important observation was that the incidence of injuries peaked in infants (44% were ≤one-year-olds) and toddlers (two and three-year-olds together represented 22% of children). Below the age of three, a child was around nine times more likely to suffer a thermal injury than in any upcoming 3-year interval (Figure 1). After the first three critical years (66% of all cases), the incidence dropped drastically and became steady.

**Figure 2:**
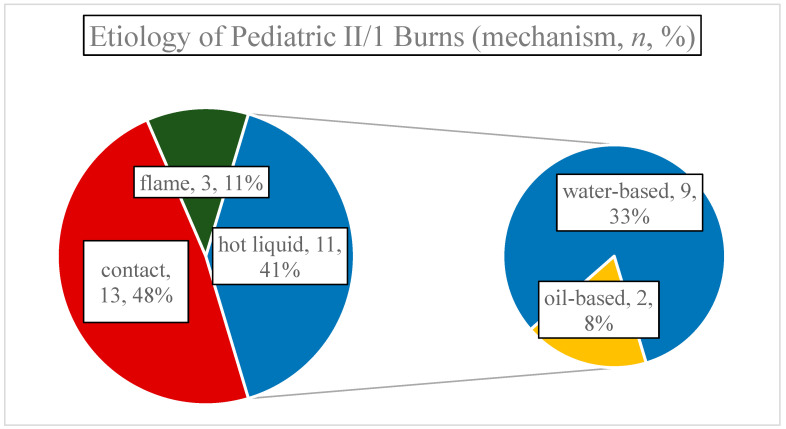
Etiology of pediatric II/1 burns (mechanism, *n*, %). Hot liquids were predominantly water-based.

Average surface of thermal wounds was 2.87% TBSA (SD:1.33, range: 1–7.5%), and etiologically distributed as follows: 13 contact- (48.15%), 11 hot liquid- (40.74%), and three flame injuries (11.11% of all cases) (Figure 2.). It is important to mention that only two patients had larger than 5% TBSA injuries, therefore, most burns were small in area. Regarding depth, a single burn was classified as mixed depth—the rest were solely II/1.

**Figure 3:**
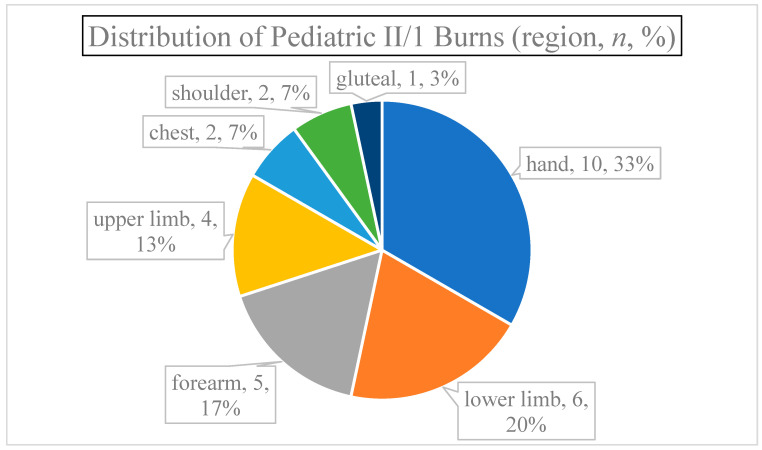
Distribution of pediatric II/1 burns (region, *n*, %). Primarily, the upper extremity was involved.

Burn localization was more varied. Six patients had multiple regions damaged (22.22% of all cases); the burn affected both the upper and lower limbs of four patients and the chest was injured along with a shoulder in two children (Figure 3). The majority of injuries involved the upper limb (70% of all cases), especially the hands (46% of each scenario), followed by the incidence on the lower extremities (23%), and lastly, the trunks (7%).

Prehospital management can be summarized by discussing the time elapsed between the injury and hospital arrival, which averaged 3.48 h, caused by a few outliers. Consequently, median and IQR25 times were both one hour with an IQR75 of 3.5 h, skewing the distribution left, and generally towards rapid transit times. Prehospital cold therapy (running water or cooling gels) was only applied in four out of five cases (81.5% received it) while any kind of analgesia was only administered for every third child (33.3% received analgesic). Absence of prehospital care negatively correlated with age (*r* = −0.349 for missing cold therapy, *r* = −0.257 for lack of analgesia, and *r* = −0.299 for the combined absence of both interventions, indicating slight to moderate negative association). Thus, most children who did not receive any intervention were below three years old. Cold treatment was only skipped in children under two years (mean age: 1.2 years) and while protocols generally lacked pain management, recipients of analgesics were also older (mean age of 5 years) compared to untreated patients (mean age of 3.1 years).

**Figure 4:**
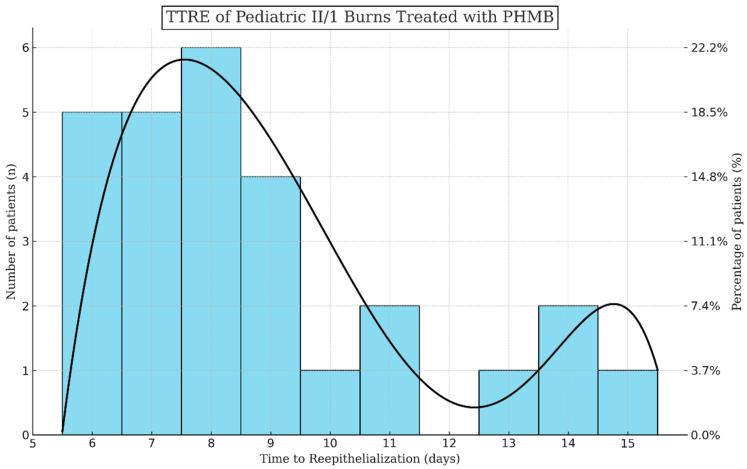
TTRE of pediatric II/1 burns treated with PHMB. The bimodal trendline illustrates the predicted TTRE distribution over the measured ones shown on the histogram.

Injuries treated with PHMB required 8.78 days to achieve complete wound closure (SD: 2.64, range: 6–15 days) while necessitating 2.37 dressing changes on average (SD: 1.6, range: 1–6 times) (Figure 4).

**Figure 5:**
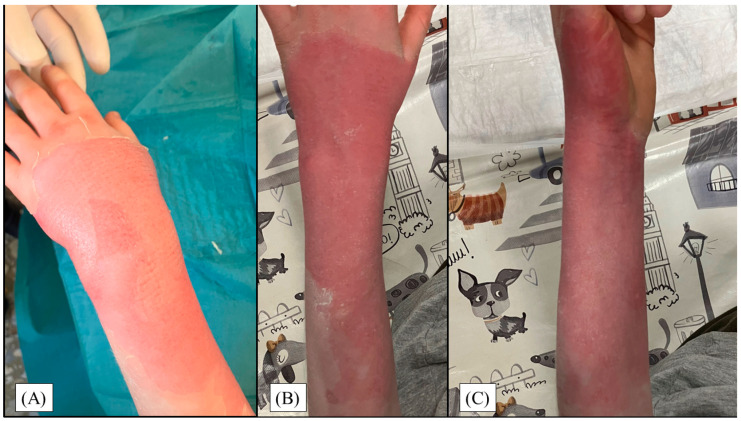
(**A**) Dorsal view of a II/1 hot liquid injury on the left forearm and hand of a 9-year-old boy after bullectomy in sedation. Control examination a week later shows complete, complication-free wound closure from dorsal (**B**) and radial aspects (**C**).

Most patients healed between the 6th and 9th days, with around 18.5% of children reepithelialized completely on each of these days. On later days, again steadily, approximately 5% of wounds closed entirely. Prolonged healing was observed in ≥5% TBSA burns, flame injuries, or cases involving the lower extremities. Visual representations of the most common wound site after injury, and subsequent to reepithelialization can be seen in Figure 5.

Distinct etiologies seemed to govern wound closure times as well, which in turn appeared to be linked to different age groups. Contact burns were most prevalent, primarily in the youngest (median age: 1, range: 1–8 years), who healed at the fastest rate (TTRE: 8.08, SD: 1.71, range: 6–11 days). Injuries via hot liquids were next in chronological and incidence order—albeit they presented the widest age range (median age: 3, range: 1–13 years) and reepithelialization distribution (TTRE: 9.36, SD: 3.17 range: 6–15 days). Least frequent were the flame wounds (median age: 8, range: 6–10) with marginally the most extended healing (TTRE: 9.67, SD: 4.04 days, range: 6–14 days). However, statistical differences were insignificant between etiologies (*p* = 0.4221 for TTRE and *p* = 0.7275 for age).

Prolonged TTRE reasonably correlated with more bandage changes (*r* = 0.706), analgesia administration (*r* = 0.699), larger burned TBSA% (*r* = 0.626), and longer LOS (*r* = 0.463) the most. Older age was also slightly associated with prolonged reepithelialization (*r* = 0.302). Remarkably, delayed intervention (*r* = 0.249), cold therapy (*r* = 0.143), and sex (*r* = 0.061) had only minimal impact on TTRE. Localization also affected wound closure times, although not significantly (*p* = 0.081). While the TTRE of the hands and the whole upper limb healed quite fast (mean TTRE: 7.60 and 7.75, respectively), when only the forearm was injured, it required 10.4 days on average to heal. The trunk and shoulders took 8 and 7.5 days, respectively, to reepithelialize, while the wound closure of the lower limbs took a noticeably longer time (average TTRE: 11.43 days).

A linear regression model developed to predict TTRE, based on factors like age, sex, TBSA affected, transport time, and the prehospital usage of cold therapy or analgesia produced the following evaluation metrics: MSE: 3.017 and R^2^: −0.358. In this case, a negative R^2^ value suggests that the simulation did not effectively capture the variability in TTRE, highlighting the complexity of predicting it based solely on the variables provided. More sophisticated models were also trained and deployed but performed inferior on this task (random forest MSE: 3.68, R^2^: −0.66; deep learning model MSE: 4.93, R^2^: −1.22).

Three instances of significantly different endpoints were found between patients who received analgesia and those who did not. Analgesia administration prolonged mean TTRE (11.33 days for those who received it (SD: 2.69, range: 8–15) as opposed to 7.5 days (SD: 1.42, range: 6–11) for those who did not, with a *p*-value of 0.0005. Analgesia increased the average number of dressing changes too (*p* = 0.0077), with 3.66 times (SD: 1.87, range: 1–6) among recipients and 1.72 times (SD: 0.95, range: 1–4) in non-recipients. However, mean TBSA also significantly differed (*p* = 0.0474), meaning patients who received analgetic had larger burns (3.61% TBSA, SD: 1.85, range: 2–8) than children who did not (2.50% TBSA, SD: 0.49, range: 2–3). 

Lastly, LOS was 0.96 days (SD: 1.73, range 0–7 days). It is important to mention that only six children spent the night at the hospitals, therefore, most of the patients could be treated in an ambulatory manner. Admissions were necessary due to ≥5% TBSA burns, mixed depth injury, or social reasons. No complications were reported or observed during the study. Summarized categorical endpoints of this study are shown in Table 1 and continuous variables are presented in Table 2.

## 4. Discussion

In this analysis, our study systematically investigated the preliminary results of the effectiveness of PHMB in treating II/1 burns among a pediatric cohort of 27 patients from two Hungarian medical centers. This research offers an insight into the demographic distribution, etiology, and treatment outcomes associated with pediatric burns, reflecting a male predominance (2 out of 3 patients were boys) and a notable susceptibility in children aged three years and below to thermal injuries (approximately 9 times higher frequency than other age groups). The incidence peak in infants and toddlers underscores a critical need for heightened awareness and preventive strategies in this vulnerable age interval. Male predisposition might be attributed to higher activity levels and risk-taking behavior. Our findings align with broader patterns observed in burn statistics worldwide [21,22,23].

These results show the predominance of contact burns, accounting for half of the injuries, followed by (mostly water-based) hot liquid and flame injuries. Despite the varying etiologies, the majority of burns were minor in terms of TBSA affected, signifying that most incidents involved limited exposure to the causative agent. In terms of initial care, the study revealed that while the majority were promptly treated with cold therapy, only a third received analgesia prior to hospital admission, suggesting an area for potential improvement in early burn care protocols. This discrepancy also raises considerations about the standardization and accessibility of prehospital care for Hungarian pediatric burn victims, particularly the youngest who are most at risk. Targeted educational programs for parents and caregivers, focusing on the most common mechanisms of burns in this population, could considerably reduce the incidence of pediatric burns.

Through our review of wound healing dynamics under PHMB treatment, an average of 8.78 days was observed for complete wound closure, with a direct correlation between longer TTRE and the number of dressing changes, as well as the percentage of TBSA affected. The low correlation (*r* = 0.249) of TTRE with the time since injury and cold therapy (*r* = 0.143) suggests, that these two factors only slightly affect wound closure. Associations between analgesics and, older age on prolonged TTRE, present intriguing avenues for further investigation into the biological and environmental factors influencing burn recovery. As most patients could be treated ambulatory, a remarkably short 0.96 days of average LOS was required to treat the children. Minimal hospitalization alleviates the distress of children associated with the medical environment, reduces their risk of hospital-acquired infections, and liberates medical staff and resources.

Analgesia administration was associated with longer TTRE and an increased number of dressing changes. These findings, while seemingly paradoxical (analgesic use most probably did not extend healing times, instead), may reflect more severe burns among recipients necessitating more intensive treatment. This possibility is highlighted by their significant difference in TTRE. Evidence implies that effective pain and stress management enhances the process of pediatric burn reepithelialization and high pain levels hinder it [24,25]. Still, for example in the cases of frequently administered opioids, careful monitoring is necessary to ensure the prevention of side effects such as nausea, vomiting, respiratory depression, or reduced GI motility, resulting in anxiety or decreased mobility, that could prolong wound closure [26,27]. These observations prompt a deeper inquiry into the relationship between Hungarian pain management strategies and wound-healing processes in pediatric burn patients, to ensure, that the currently administered analgesics do not delay reepithelialization.

The absence of complications reported in our study is encouraging but also warrants a broader review of the long-term outcomes of PHMB-treated burns. It is necessary to investigate how these children fare in terms of scar formation, functional mobility, and psychological impact compared to those treated with traditional methods.

When juxtaposed with randomized controlled trials (RCT) investigating SSD and other alternatives applied to similar pediatric burns, the attributes of PHMB—broad-spectrum antimicrobial activity, low cytotoxicity, and a favorable profile for wound healing—suggest its potential as a superior option in specific contexts of pediatric burn care [6,28]. In contrast, SSD needed 14.74 days using a weighted average (mean ranges: 12.75–16 days) when applied to similar-depth injuries- although, the authors did not report TBSA in these trials [29,30]. Hydrofiber dressings [29], hydrogels [30], biosynthetic porcine xenografts, and hydrocolloid coverings [31] required 14.0, 12.42, 12.24, and 11.21 days for reepithelialization on average, respectively. Zinc-hyaluronan gel achieved slightly superior results to PHMB, requiring only 7.9 days for wound closure, however, it was only applied to the facial region, which has enhanced capillarization and regenerative capabilities [32].

Regarding cost-effectiveness, we evaluated the prices of SSD and PHMB in our country, at the time of writing (April 2024)—although, costs and availability may vary with time and nation. Despite initial expenses being lower for SSD (0.18 vs. 0.41 USD/g), it has a higher dressing reapplication rate. An RCT found that SSD necessitated 5.13 dressing changes to close 1.92% TBSA burns [33]. When only comparing the cost of the interventions utilizing a symbolic point-sized II/1 burn (a standard burn area unit requiring 1 g of cream or gel to heal per application), the total SSD cost was 0.92 USD while PHMB was 0.97 USD. However, dressing changes require additional resources, like bandages, and are often performed under analgesia or anesthesia, which further increases expenditure per reapplication, and might highly differ based on hospital protocols. Another RCT showed that larger II/1 burns (10.13% TBSA) required even more reapplications (9.27 times) of SSD [34]. This seems to be true for PHMB as well (TTRE was 8.32 days and dressing changes were 2.12 on average for patients with <5% TBSA burns, while 5–10% TBSA injuries required 14.50 days and 5.5 reapplications of PHMB for wound closure), further complicating calculations. A third trial treated the wounds openly with SSD, necessitating considerably more dressing changes (65.53 times to close an average of 9.39% TBSA) than when combining the cream with bandages [35]. The broader adoption of PHMB requires further investigation, particularly in direct comparison to these traditional agents, to understand its place fully within the spectrum of burn treatments.

Limitations of our study, including its small sample size, short follow-up period, and observational design, necessitate cautious interpretation of the results. Despite being multi-centric, this study only evaluated patients from Hungary, therefore, the generalization of results to different healthcare environments and populations may be limited. As prehospital care was inconsistent due to the different transportation methods and the varying burn care knowledge of the first aiders, recovery outcomes might be biased. Due to the retrospective nature of the study, many important factors could not be appropriately analyzed, such as pain and satisfaction levels, which may ultimately affect the TTRE. Complexities of predicting TTRE were highlighted by the challenges in developing an effective linear regression model—as evidenced by negative R-squared values in our predictive analyses -, suggesting, that a critical variable is still absent from the evaluation. The potential for selection bias and the inability to establish a TTRE-predicting model highlights the need for larger, RCTs to validate our findings and explore the mechanistic underpinnings of PHMB in burn management. Direct correlation of the results to established interventions is challenging, due to the lack of a control group.

Consequently, future research should aim to expand the demographic and etiological spectrum of pediatric burn patients studied, incorporating a broader range of burn severities in addition to sizes and therapeutic modalities. Investigating the long-term outcomes of PHMB treatment, including scar formation and functional impairment, will be crucial in systematically evaluating its role in pediatric burn care.

This study sheds light on significant aspects of pediatric burn injuries and their management, presenting PHMB as a promising agent in the therapeutic arsenal. It also lays bare the complexities of Hungarian burn care in children, urging further exploration into the intricacies of wound healing, optimized treatment protocols including novel interventions, and prehospital care standardization—which may help reveal patterns in other healthcare environments as well. Through continued research and innovation, we can aspire to significantly improve outcomes for pediatric burn victims, paving the way for more effective, individualized approaches to burn prevention and care.

## 5. Conclusions

Our study underscores the potential of PHMB in pediatric burn care, showcasing its swift wound closure capabilities combined with minimal need for dressing changes and remarkably short hospital stays. PHMB distinguished itself by facilitating healing without complications, however, the research beckons further exploration into the long-term impact of PHMB compared to conventional methods.

This evaluation also unearthed stark demographic trends in Hungary: children under three faced a nine times higher risk of burn injuries while receiving the least amount of analgesia and cold therapy, compared to any other three-year intervals. Advocacy for comprehensive preventive and educational initiatives emerges, aimed at arming caregivers and responders with essential skills for immediate burn treatment.

## Figures and Tables

**Table 1:** Descriptive statistics of discrete outcomes regarding patient and II/1 burn characteristics, which were treated with PHMB.

Variable (*N* = 27)	Category	Count (*n*)	Percentage (%)
Sex	Male	18	66.67
	Female	9	33.33
Cold therapy?	Yes	22	81.48
	No	5	18.52
Analgesia?	No	18	66.67
	Yes	9	33.33
Burn depth	II/A	26	96.30
	II/A-B (Mixed)	1	3.70
Etiology	Contact	13	48.15
	Hot liquid	11	40.74
	Flame	3	11.11
Complications	No	27	100.00

**Table 2:** Descriptive continuous variable statistics for patient characteristics and PHMB-treated II/1 burns, using medians and IQRs due to non-normality. Means and additional metrics are also provided, accounting for significant left skews, especially in LOS.

Variable (*N* = 27)	Median	IQR	IQR25	IQR75	Mean	SD	Min	Max
Age (years)	2.0	5.5	1.0	6.5	3.74	3.54	1	13
Time since injury (hours)	1.0	2.5	1.0	3.5	3.48	4.73	1	17
TBSA (%)	2.5	1.0	2.0	3.0	2.87	1.33	1	8
Dressing change (*N*)	2.0	2.0	1.0	3.0	2.37	1.60	1	6
TTRE (days)	8.0	2.5	7.0	9.5	8.78	2.64	6	15
LOS (days)	0.0	0.0	0.0	0.0	0.96	2.10	0	7

## Data Availability

All data is contained within the article.
